# Effect of paricalcitol on renin and albuminuria in non-diabetic stage III-IV chronic kidney disease: a randomized placebo-controlled trial

**DOI:** 10.1186/1471-2369-14-163

**Published:** 2013-07-26

**Authors:** Thomas Larsen, Frank H Mose, Jesper N Bech, Erling B Pedersen

**Affiliations:** 1 Department of Medical Research and Medicine, Holstebro Hospital, Laegaardvej 12, Holstebro 7500, Denmark; 2 University of Aarhus, Aarhus C 8000, Denmark

**Keywords:** Albuminuria, Chronic kidney disease, Nitric oxide, Vitamin D, Renin-angiotensin system

## Abstract

**Background:**

Vitamin D receptor activators reduce albuminuria, and may improve survival in chronic kidney disease (CKD). Animal studies suggest that these pleiotropic effects of vitamin D may be mediated by suppression of renin. However, randomized trials in humans have yet to establish this relationship.

**Methods:**

In a randomized, placebo-controlled, double-blinded crossover study, the effect of oral paricalcitol (2 μg/day) was investigated in 26 patients with non-diabetic, albuminuric stage III-IV CKD. After treatment, plasma concentrations of renin (PRC), angiotensin II (AngII) and aldosterone (Aldo) were measured. GFR was determined by ^51^Cr-EDTA clearance. Assessment of renal NO dependency was performed by infusion of NG-monomethyl-L-arginine (L-NMMA). Albumin excretion rate (AER) was analyzed in 24-h urine and during ^51^Cr-EDTA clearance.

**Results:**

Paricalcitol did not alter plasma levels of renin, AngII, Aldo, or urinary excretion of sodium and potassium. A modest reduction of borderline significance was observed in AER, and paricalcitol abrogated the albuminuric response to L-NMMA.

**Conclusions:**

In this randomized, placebo-controlled trial paricalcitol only marginally decreased AER and did not alter circulating levels of renin, AngII or Aldo. The abrogation of the rise in albumin excretion by paricalcitol during NOS blockade may indicate that favourable modulation of renal NO dependency could be involved in mediating reno-protection and survival benefits in CKD.

**Trial registration:**

ClinicalTrials.gov identifier: NCT01136564

## Background

Vitamin D receptor activators (VDRAs) have been used in the management of secondary hyperparathyroidism in chronic kidney disease (CKD) for several decades [1]. Selective VDRAs such as paricalcitol effectively suppress PTH secretion, but have less impact on gastrointestinal calcium and phosphate uptake compared with calcitriol. Recently, it has been shown that VDRAs reduces albuminuria in CKD and may slow down progression of renal damage and dysfunction [2-4]. Conclusions from animal studies strongly suggest that vitamin D’s proposed reno-protective properties may be mediated by suppression of the renin-angiotensin system (RAS) [5-7]. Additionally, calcitriol has been shown to reduce renal inflammation [8], and preserve the glomeruli slit diaphragm by abrogating loss of nefrin [9]. The integrity of the glomerular filtration barrier is highly dependent on nitric oxide (NO) [10], and acute blockade of the NO synthase (NOS) by NG-monomethyl-L-arginine (L-NMMA) provokes albuminuria in humans [11]. Animal studies indicate that modulation of the NO system may represent another pathway by which vitamin D reduces albuminuria [12,13].

Despite pharmacological advances in the treatment of renal disease, the progressive nature of CKD still warrants new strategies to preserve kidney function. VDRAs are novel candidates, although their reno-protective properties in man remain to be elucidated. In the current randomized, placebo-controlled, double-blind crossover trial, we wanted to test the hypothesis that paricalcitol decreases albuminuria in CKD by suppressing plasma renin. Moreover, we hypothesized that paricalcitol modulates renal NO bioavailability. This was investigated by employing acute NOS inhibition by L-NMMA. Hemodynamic and non-hemodynamic effects of paricalcitol were assessed by measurement of 24-hour ambulatory BP (24-h BP), arterial stiffness, GFR, albumin excretion rate, renal sodium and potassium excretion, and plasma concentrations of angiotensin II (AngII), aldosterone (Aldo), vasopressin (AVP), brain natriuretic peptide (BNP) and fibroblast growth factor 23 (FGF23).

## Methods

### Participants

#### Recruitment

Subjects were recruited from the nephrology outpatient unit at Holstebro Hospital, Denmark between June 2010 and August 2011. *Inclusion criteria* were age >18 years, stage III-IV CKD (eGFR: 15–59 ml/min) and micro- or macroalbuminuria (u-alb >30 mg/l). *Exclusion criteria* were diabetes mellitus (hbA1c ≥6.5%), cardiac arrhythmias, malignant disease, immunosuppressive treatment, alcohol abuse (>24 grams per day), p-albumin <25 mmol/l, hypercalcemia (p-Ca^++^ >1.32 mmol/l) and office BP >170/105 mmHg. *Withdrawal criteria* were development of exclusion criteria, medication changes, withdrawal of consent and poor compliance. *Sample size* was calculated using PRC as primary endpoint. With a significance level of 5% and an 80% power to detect a 10 pg/ml difference in PRC, a total of 24 subjects were needed in this cross-over study.

### Ethics

The study protocol was approved by the Danish Medicines Agency (Eudract no 2009-017619-14), the Regional Committee on Biomedical Research Ethics (journal no M-20090236), and The Danish Data Protection Agency. The trial was registered at http://www.clinicaltrials.gov (ClinicalTrial identifier: NCT01136564) and carried out in accordance with the Declaration of Helsinki. Written informed consent was obtained from each patient.

### Design

This was a randomized, placebo-controlled, double blinded, crossover study. Participants were consecutively allocated to treatment via computer-generated randomization using blocks of six, and received paricalcitol capsules and matching placebo in random order. After inclusion, each patient entered a four-week run-in phase where any treatment involving vitamin D or RAS inhibitors were discontinued. If needed, amlodipine, a diuretic, metoprolol or minoxidil was added to control hypertension. No changes in BP medication were made after the run-in phase of the study. Patients took the study drug orally as two capsules daily for two periods of six weeks with a two-week intercalated washout period. Safety visits were conducted every two weeks to monitor p-Ca^++^, BP, compliance and potential side effects.

### Study drug

Paricalcitol (1 μg per capsule) and placebo (Abbott Laboratories, IL, USA) were taken orally as two capsules per day. The non-selective NOS inhibitor, L-NMMA (Bachem, Germany), was dissolved in isotonic saline solution.

### Experimental procedure

Measurements were performed at the end of each six week treatment period of which the last four days encompassed intake of a standardized diet. The diet was composed to fit energy requirements determined by weight and level of physical activity at work, leisure and workout. If the estimated energy requirement exceeded 9,500 kJ/day a large diet was given (11,000 kJ/day). Otherwise a small diet (8,000 kJ/day) was given. The diet consisted of three main and three in-between meals per day, and contained 55% carbohydrates, 30% fat and 15% protein. The sodium content was 130 mmol/day in the larger diet and 95 mmol/day in the smaller. Fluid intake was 35 ml water/kg/day, and no other liquids were allowed during those four days.

An outline of each of the two examination days is given in Additional file 1: Table S1. At 8 AM two indwelling catheters for blood sampling and infusion of ^51^Cr-EDTA and L-NMMA were placed in cubital veins, one in each arm. Subjects were water loaded with oral tap water 175 ml every 30 minutes, and urine was collected by voiding in the standing or sitting position. Otherwise subjects were kept in supine position in a quiet, temperature-controlled room (22-25°C). ^51^Cr-EDTA was adjusted for weight and renal function, and administered as a priming dose at 8:30 AM followed by sustained infusion. Urine collected the first 60 minutes after priming dose was not analyzed. At 11 AM an L-NMMA bolus injection of 4.5 mg/kg was given, followed by continuous infusion at a rate of 3.0 mg/kg/hr for 60 minutes. Blood and urine samples were collected every 30 min from 9:30 AM to 1 PM, and analyzed for ^51^Cr-EDTA, electrolytes, albumin and osmolality. The three clearance periods from 9:30 AM to 11 AM were pooled for analysis and used to define baseline. These were followed by four 30 min clearance periods. For determining hormone levels, blood samples were drawn at 11 AM (baseline), at noon (after 60 min of study drug infusion), and at 1 PM (60 min after cessation of infusion). Applanation tonometry was performed at 10.40 AM (baseline) and 11.40 AM.

### Effect variables

The prespecified primary end point was plasma renin concentration (PRC). Secondary effect variables were urinary albumin excretion rate (AER), carotid-femoral pulse wave velocity (PWV), Augmentation Index (AIx), GFR, fractional excretion of sodium (FE_Na_) and potassium (FE_K_) urinary excreation of aquaporin 2 channels (AQP2) and epithelial sodium channels (ENaC_γ_), FGF23, and AngII, Aldo, BNP and AVP at baseline and during NOS blockade.

### Biochemical analyses

Blood samples for determination of vasoactive hormones were taken on ice and centrifuged for 15 min at 3500 rpm and kept frozen at −80°C or −20°C until assayed all at once. *PRC* was determined using an immunoradiometric assay from CIS Bio International, Gif-Sur-Yvette Cedex, France. Minimal detection level was 1 pg/ml. The coefficients of variation were 0.9-3.6% (intra-assay) and 3.7-5.0% (inter-assay) in the range of 4–263 pg/ml. *AngII* was extracted from plasma with C_18_ Sep-Pak (Water associates, Milford, MA, USA), and subsequently determined by radioimmunoassay (RIA) as previously described [14]. The antibody was obtained from the Department of Clinical Physiology, Glostrup Hospital, Denmark. Minimal detection level was 1.5 pg/ml. The coefficients of variation were 12% (inter-assay) and 8% (intra-assay). *Aldo* was determined by RIA using a kit from Demeditec Diagnostics GmbH, Germany. Minimal detection level was 25 pmol/l. The coefficients of variation were 6.7-10.4% (inter-assay) and 7.5-9.5% (intra-assay). *AVP* was extracted as Ang II and determined by radioimmunoassay^14^. The antibody against AVP was a gift from Professor Jacques Dürr, Miami, Fl, USA. Minimal detection level was 0.2 pmol/L. The coefficients of variation were 13% (inter-assay) and 9% (intra-assay).

Intact plasma levels of *FGF23* were determined by a sandwich ELISA (Immutopics Inc., CA, USA). The coefficients of variation were 6% (inter-assay) and 4% (intra-assay). Commercial chemiluminescens immunoassays were used to analyze plasma concentrations of *25(OH)D2 + D3* (Liaison, DiaSorin Inc., Saluggia, Italy) and *BNP* (Architect, Abbott Laboratories, IL, USA).

Urine samples were kept frozen at −20°C until assayed. *u-AQP2* was measured by radioimmunoassay as previously described, and antibodies were raised in rabbits to a synthetic peptide corresponding to the 15 COOH-terminal amino acids in human AQP-2 to which was added an NH_2_-terminal cystein for conjugation and affinity purification. Minimal detection level was 0.15 ng/tube. The coefficients of variation were 11.7% (inter-assay) and 5.9% (intra-assay) [15]*. u-ENaC*γ was measured by radioimmunoassay as previously described, and antibodies were raised in rabbits. Minimal detection level was 48 pg/tube. The coefficients of variation were 14% (inter-assay) and 7.4% (intra-assay) [16].

Plasma and urinary osmolality were measured by freezing-point depression (Advanced Model 3900 multisampling osmometer). Routine blood and urine chemistry including a complete blood count, basic metabolic panel, cholesterol, ionized calcium, iPTH, phosphate, 25(OH)D_2+3_ as well as urinary albumin and calcium concentrations, were immediately assayed at the Department of Clinical Biochemistry.

### Calculations

Free water clearance (C_H2O_) was determined according to the formula C_H2O_ = urine output (V) - osmolar clearance (C_OSM_). FE_Na_ and FE_K_ were calculated according to the formula FE_x_ = (u ‒ X * V/p ‒ X)/GFR during the clearance experiment and in 24-h urine according to the formula FE_x_ = (u ‒ X * p ‒ Crea)/(p ‒ X * u ‒ Crea).

### Renal function

Glomerular filtration rate was measured using constant infusion clearance technique with ^51^Cr-EDTA as a reference substance. Estimate GFR (eGFR) was calculated using the MDRD formula.

### Ambulatory BP measurement

24-h BP was measured (Kivex TM-2430, Hoersholm, Denmark). BPs were recorded in the non-dominant arm every 15 min during daytime and every 30 min overnight.

### Applanation tonometry

Carotid-femoral PWV and radial pulse wave analysis (PWA) were obtained by a trained technician using applanation tonometry (SphygmoCor CPV system®, Atcor Medical, Sidney, Australia). Only duplicate recordings that met the minimum quality requirements were included in the final analysis. Brachial BP used for PWA was recorded by a semiautomatic, oscillometric device (Omron 705IT, Japan). If the difference within a duplicate BP recording exceeded 7 mmHg, the BP measurement was repeated.

### Statistical analysis

Differences between placebo and paricalcitol were compared using Student’s paired *t*-test for parametric data, and Wilcoxon’s Signed Rank test for non-parametric data. Estimates are given as means ± SD unless stated otherwise. The effect of L-NMMA on repeatedly measured variables was assessed by Friedman’s two-way ANOVA. The effect of paricalcitol on the L-NMMA response was analyzed by a general linear model (GLM) for repeated measures with paricalcitol/placebo and time as within-subject factors. Spearman’s rho was used for correlation analyses. Statistical significance was defined as p < 0.05. Statistical analyses were performed using PASW version 18.0.0 (SPSS Inc.; Chicago, IL, USA).

## Results

### Demographics

**Figure 1:**
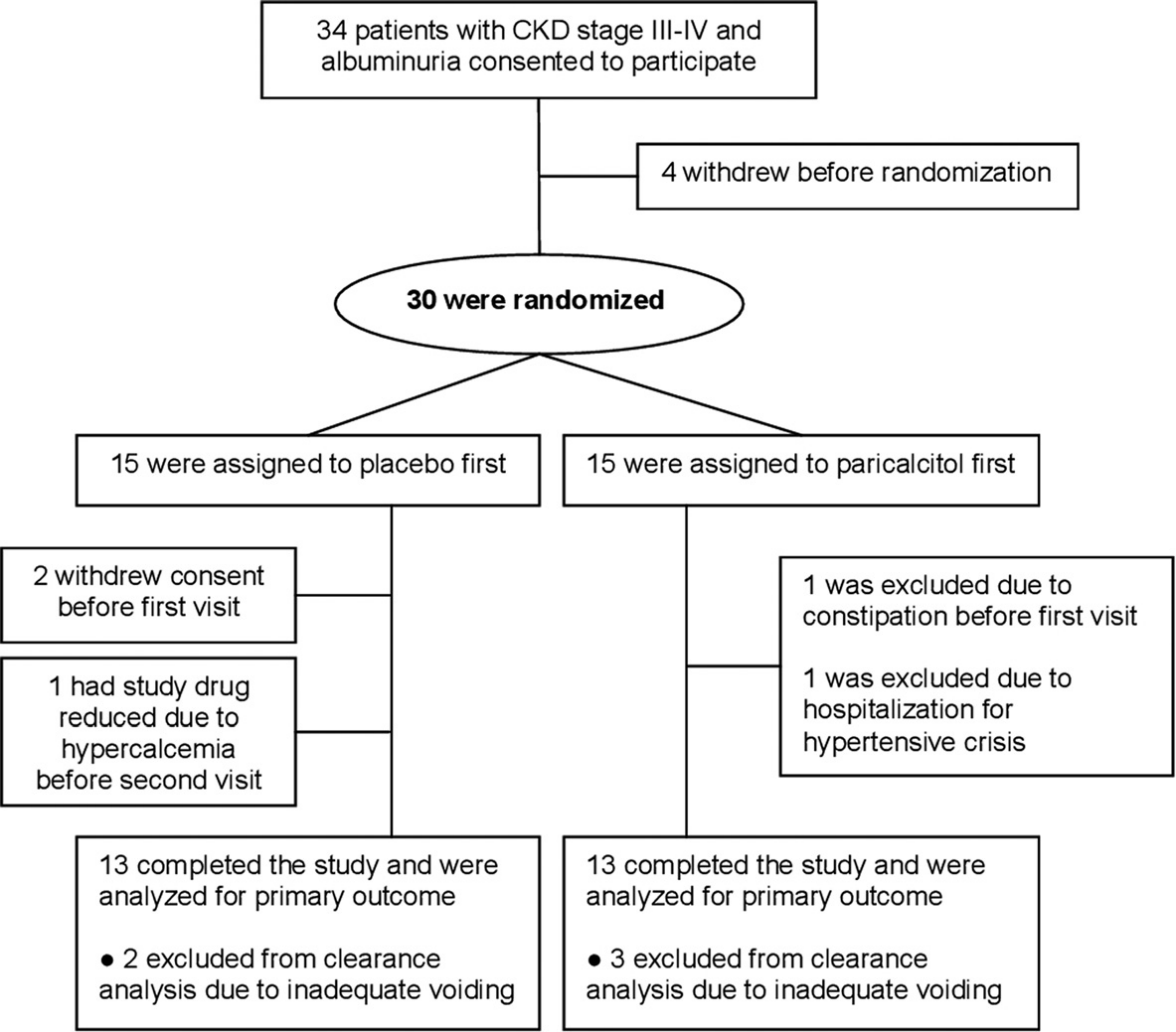
Flowchart.

Recruitment and demographics are given in Figure 1 and Table 1, respectively. Compliance with study medication assessed by pill count was 99.7% for placebo and 99.4% for paricalcitol. Five males were excluded from analysis of clearance related variables due to inadequate ability to empty the bladder. These subjects did not differ from the overall study population regarding age, eGFR and baseline albuminuria.

**Table 1:** Patient demographics (n = 26)

**Age**, years (±SD)	61 ± 9
**Male sex,** no (%)	19 (73)
**Smokers**, no (%)	13 (50)
**Body mass index**, kg/m^2^ (±SD)	26.7 ± 4.6
**Office blood pressure**, mmHg (±SD)	136/83 ± 13/9
**Medication**, no (%)	
Calcium channel blockers	20 (77)
Alpha blockers	1 (4)
Beta blockers	16 (62)
Minoxidil	7 (27)
Loop diuretics	16 (62)
Thiazide diuretics	3 (12)
Spironolactone	2 (8)
Allopurinol	3 (12)
Statins	16 (62)
**Number of antihypertensive agents** (±SD)	2.5 (1.3)
**Routine Biochemistry** (±SD)	
eGFR, ml/min/1.73 m^2^ (MDRD)	26 ± 11
p-Creatinine, μmol/l	231 ± 78
p-Ca^++^	1.22 ± 0.05
p-Phosphate, mmol/l	1.19 ± 0.27
p-Cholesterol (total), mmol/l	5.0 ± 1.2
p-Hemoglobin, mmol/l	8.3 ± 0.8
p-Albumin, g/l	43 ± 3
u-Albumin, mg/l	169 [Interquartile range: 59;489]
P25(OH)D_2+3,_ nmol/l	56 ± 21
**Primary kidney disease**, no (%)	
Glomerulonephritis	6 (23)
Polycystic kidney disease	3 (12)
Chronic interstitial nephritis	2 (8)
Unknown*	15 (58)

*Renal biopsy deferred due to advanced stage of kidney disease on admission, reduced kidney size on radiologic examination and absence of nephrotic syndrome.

### The impact of paricalcitol on calcium metabolism and FGF23

Paricalcitol reduced PTH and marginally increased concentrations of phosphate and Ca^++^ as well as 24-h urinary calcium excretion (Table 2). One patient in the paricalcitol group developed asymptomatic hypercalcemia after four weeks of treatment (Ca^++^: 1.43 mmol/l), and the study drug was reduced to one capsule daily with normalization of p-Ca^++^ at follow-up two weeks later. Paricalcitol increased FGF23 by 46% (95%CI: [21;71], p = 0.001). A weak positive correlation between changes in FGF23 and changes in p-phosphate was observed (r = 0.40; p = 0.04). Likewise, changes in FGF23 correlated negatively with changes in PTH (r = −0.42; p = 0.03).

**Table 2:** Effect of paricalcitol on calcium metabolism, blood pressure and 24-h albumin excretion in patients with stage III-IV CKD and albuminuria (n = 26)

	**Placebo**	**Paricalcitol**	**P value**
*Calcium and phosphate metabolism*			
p-iPTH (pmol/l)	11.80 [8.82;14.93]	6.44 [3.11;8.78]	<0.001
p-Ca^++^ (mmol/l)	1.22 [1.16;1.25]	1.23 [1.19;1.28]	0.01
p-phosphate (mmol/l)	1.18 [0.97;1.35]	1.29 [1.10;1.59]	0.02
p-ALP (U/l)	66 [58;91]	65 [51;82]	<0.001
p-25(OH)D (nmol/l)	58 [39;72]	59 [50;69]	0.26
p-FGF23 (pg/ml)	28.5 [19.7;46.7]	32.3 [27.2;67.1]	0.002
u-Ca (mmol/24-h)	0.78 [0.39;2.03]	1.24 [0.66;3.04]	0.006
u-Ca/cr (mmol/mmol)	0.06 [0.03;0.15]	0.12 [0.06;0.22]	0.005
*24-h ambulatory blood pressure and arterial stiffness*			
24-h SBP (mmHg)	133 ± 11	133 ± 10	0.92
24-h DBP (mmHg)	81 ± 7	81 ± 7	0.94
24-h heart rate (bpm)	67 ± 9	68 ± 9	0.15
PWV (m/s)	7.9 ± 1.3	7.6 ± 1.3	0.21
AIx@75 (%)	21 ± 13	19 ± 11	0.30
Central SBP (mmHg)	132 ± 12	130 ± 13	0.34
Central DBP (mmHg)	82 ± 7	82 ± 6	0.97
*Creatinine clearance and albumin excretion in 24-h urine*			
Urine output (ml/24-h)	2805 [2430;3160]	2794 [2270;3210]	0.43
Creatinine clearance (ml/min/1.73 m^2^)	36 [28;51]	32 [27;46]	0.005
u-alb (mg/24-h)	665 [80;2598]	590 [146;2025]	0.12
UACR (mg/g)	507 [80;1523]	388 [104;1109]	0.34

Values are presented as medians with 25 and 75 percentiles in brackets, or as means ± SD. *iPTH* Intact parathyroid hormone, *Ca*^++^ Ionized calcium, *ALP* Alkaline phosphatase 25(OH)D, 25-hydroxyvitamin D; FGF23, fibroblast growth factor 23; u-Ca/cr, urinary calcium-creatinine ratio; PWV, carotid-femoral pulse wave velocity; AIx@75, central augmentation index standardized to a heart rate of 75 beats per minute (bpm); UACR, urinary albumin-creatinine ratio. P values represent the probability of a difference between treatments.

### The impact of paricalcitol on blood pressure and arterial stiffness

**Figure 2:**
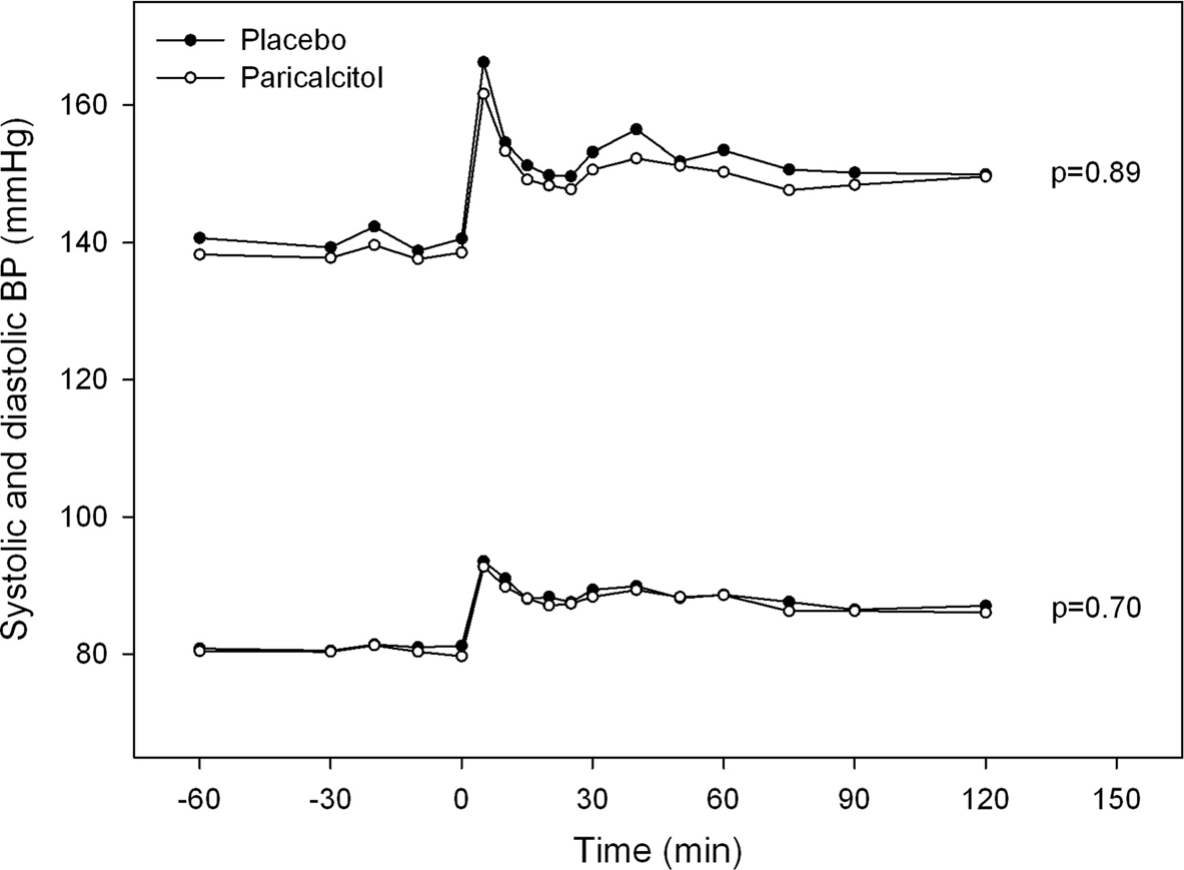
**Effect of L-NMMA on systolic and diastolic blood pressure (BP).** L-NMMA bolus injection was administered at 0_min_, and sustained infusion continued until 60_min_. P-value represents the effect of paricalcitol versus placebo assessed by a general linear model for repeated measures (n = 26).

Ambulatory BP and heart rate were not affected by paricalcitol (Table 2). Similarly, no difference in PWV, AIx and central BP estimated by applanation tonometry were observed between placebo and paricalcitol. L-NMMA infusion elicited a temporary increase in both systolic and diastolic BP (Figure 2) as well as PWV (placebo: 0.4 ± 1.0 m/s; paricalcitol: 0.5 ± 0.8 m/s) and AIx (placebo: 3.4 ± 2.7%; paricalcitol: 3.9 ± 5.5%). These effects were not altered by paricalcitol.

### The impact of paricalcitol on albuminuria before and after NOS inhibition

**Figure 3:**
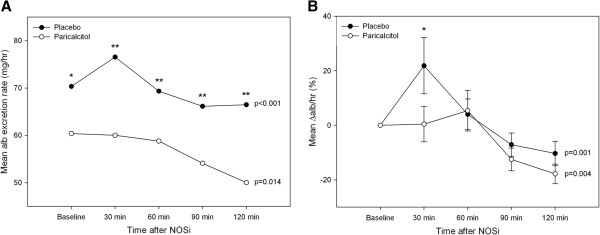
**Effect of L-NMMA on albumin excretion rate. A)** Mean albumin excretion rate in placebo (closed circles) and paricalcitol (open circles) at baseline (average for 3 periods of 30 minutes) and during nitric oxide synthase inhibition (NOSi). **B)** Mean changes in albumin excretion rate ± SEM at baseline and during NOSi. L-NMMA was continuously infused for 60 minutes. Within differences in each group were tested by Friedman’s two-way ANOVA, and comparisons between placebo and paricalcitol were made for each point in time by Wilcoxon’s signed rank test (*p < 0.05, **p < 0.005) (n = 21).

There was no significant difference in 24-h urinary albumin excretion between paricalcitol and placebo (−7% [95%CI:-20;7] p = 0.12, Table 2). However, during the 90-min baseline period of the clearance experiment (Additional file 1: Table S1) AER was significantly reduced by 19% [95%CI:-31;-8] (p = 0.003) and UACR by 20% [95%CI:-30;-11] (p < 0.001) compared with placebo. Figure 3 illustrates the changes in AER related to L-NMMA infusion. During the first 30 minute period after L-NMMA infusion was initiated, AER increased 22% [95%CI:1;43] when patients had received placebo. On the contrary, L-NMMA did not cause a rise in AER after paricalcitol treatment (0% [95%CI:-13;14], p = 0.02 between treatments).

### The impact of paricalcitol on kidney function before and after NOS inhibition

Compared with placebo, plasma creatinine increased from 223 ± 88 to 243 ± 97 μmol/l (p = 0.004) after paricalcitol. Estimated GFR (MDRD) decreased by 9 ± 11% from 29 ± 13 to 26 ± 12 ml/min (p < 0.001), 24-h urine creatinine clearance by 7 ± 12% from 40 ± 17 to 37 ± 16 ml/min/1.73 m^2^ (p = 0.002) (Table 2), and ^51^Cr-EDTA clearance by 7 ± 14% from 26 ± 13 to 24 ± 12 ml/min/1.73 m^2^ (p = 0.003). During the subsequent infusion of L-NMMA, ^51^Cr-EDTA clearance, urine output and C_H2O_ were consistently lower after paricalcitol treatment compared with placebo. However, the response to L-NMMA was not affected by paricalcitol (Table 3).

**Table 3:** The effect of L-NMMA on GFR and renal handling of sodium and potassium after six weeks of paricalcitol treatment in patients with stage III-IV CKD and albuminuria (n = 21)

	***Baseline***	***30 min***	***60 min***	***90 min***	***120 min***	***P***_***Within***_	***P***_***Between***_
**GFR (**^**51**^**Cr-EDTA clearance)**							
Placebo	26.3 ± 13.3	22.8 ± 10.9	24.2 ± 11.1	26.0 ± 11.7	27.8 ± 11.5	0.68	0.31
Paricalcitol	23.6 ± 11.5*	20.3 ± 9.7	22.5 ± 10.9	22.5 ± 12.0	23.9 ± 12.8	0.89	
**Urine output (ml/min)**							
Placebo	4.5 ± 2.1	3.1 ± 1.3	3.4 ± 1.2	3.6 ± 1.1	4.0 ± 1.8	0.02	0.15
Paricalcitol	3.4 ± 1.5*	2.4 ± 0.9	2.8 ± 1.2	3.1 ± 1.1	3.7 ± 1.5	0.01	
**Free water clearance (ml/min)**							
Placebo	1.7 ± 1.7	1.6 ± 1.4	1.1 ± 0.9	1.3 ± 0.9	1.5 ± 1.0	0.65	0.16
Paricalcitol	0.8 ± 1.0*	0.8 ± 1.0	0.7 ± 0.7	1.0 ± 0.9	1.3 ± 0.9	0.27	
**FE**_**Na **_**(%)**							
Placebo	5.9 ± 4.3	4.7 ± 3.4	4.4 ± 3.1	4.2 ± 2.8	4.1 ± 2.7	0.37	0.54
Paricalcitol	5.9 ± 4.5	4.4 ± 3.7	4.4 ± 3.4	4.6 ± 4.1	4.6 ± 4.0	0.73	
**FE**_**K **_**(%)**							
Placebo	85 ± 95	67 ± 70	62 ± 62	58 ± 51	55 ± 45	0.61	0.74
Paricalcitol	92 ± 96	71 ± 65	67 ± 58	66 ± 55	63 ± 50	0.63	
**u-AQP2 (ng/hr)**							
Placebo	99 ± 37	71 ± 29	77 ± 34	80 ± 37	87 ± 51	0.16	0.67
Paricalcitol	100 ± 35	71 ± 30	74 ± 29	75 ± 32	75 ± 31	0.03	
**u-ENaC**_**γ **_**(ng/hr)**							
Placebo	9.6 ± 7.7	-	8.8 ± 7.8	-	9.8 ± 9.0	0.92	0.32
Paricalcitol	10.3 ± 5.3	-	8.2 ± 2.3	-	8.8 ± 3.7	0.21	

Values are means ± SD. L-NMMA, NG-monomethyl-L-arginine; FE_Na_, fractional excretion of sodium; FE_K_, fractional excretion of potassium; AQP2, aquaporin 2 channels; ENaC_γ_, gamma fraction of epithelial sodium channels. L-NMMA infusion was sustained for 60 minutes. P_within_ represents the probability of an effect of NOS inhibition after each treatment assessed by one-way ANOVA. P_between_ represents the probability that paricalcitol alters the response to L-NMMA assessed by a general linear model for repeated measures with treatment as factor.

* = p < 0.05 vs. placebo at baseline.

### The impact on paricalcitol on vasoactive hormones and renal handling of sodium and potassium

Tables 3 and 4 summarize the effects of paricalcitol on RAS and related variables at baseline and in relation to L-NMMA infusion. Whereas plasma levels of renin and AngII were not affected by paricalcitol, Aldo tended to increase (Table 4). A decrease in plasma potassium was also observed, and the relative changes in Aldo correlated positively with relative changes in FE_K_ (r = 0.65, p = 0.002). Paricalcitol did not alter FE_Na_ or the urinary excretion of ENaCγ and AQP2 (Table 3).

**Table 4:** The effect of L-NMMA on components of the renin-angiotensin system and plasma levels of sodium and potassium after six weeks of paricalcitol treatment (n = 26)

	***Baseline***	***60 min***	***120 min***	***P***_***Within***_	***P***_***Between***_
**PRC (pg/ml)**					
Placebo	10.4 [6.1;23.6]	10.1 [6.1;18.8]	8.2 [6.8;17.1]	0.02 <0.01	0.31
Paricalcitol	10.9 [6.4;25.2]	8.4 [5.7;18.3]	8.9 [6.5;19.3]		
**p-AngII (pg/ml)**					
Placebo	5.0 [8.0;12.0]	6.5 [4.0;12.0]	7.5 [5.0;9.0]	0.01	0.17
Paricalcitol	8.0 [5.0;15.0]	7.0 [5.0;12.0]	8.0 [4.5;12.0]	<0.01	
**p-Aldo (pmol/l)**					
Placebo	287 [152;607]	376 [180;663]	302 [161;520]	<0.01	0.24
Paricalcitol	335 [187;619]	346 [200;742]	282 [165;590]	<0.01	
**p-K (mmol/l)**					
Placebo	4.0 [3.7;4.4]	3.9 [3.7;4.4]	3.9 [3.7;4.4]	0.96	0.19
Paricalcitol	3.8 [3.6;4.2]*	3.7 [3.6;4.2]	3.8 [3.6;4.2]	0.80	
**p-Na (mmol/l)**					
Placebo	138 [136;140]	138 [136;138]	137 [135;140]	0.76	0.33
Paricalcitol	138 [136;140]	138 [136;140]	138 [136;140]	0.33	
**p-BNP (pmol/l)**					
Placebo	8.1[4.4;19.5]	-	-		
Paricalcitol	7.5[4.8;14.3]	-	-		
**p-AVP (pg/ml)**					
Placebo	0.5 [0.4;0.7]	-	-		
Paricalcitol	0.5 [0.4;0.7]	-	-		

Values are medians with 25 and 75 percentiles in brackets. L-NMMA, NG-monomethyl-L-arginine; PRC, plasma renin concentration; AngII, angiotensin II; Aldo, aldosterone; K, potassium; Na, sodium; BNP, brain natriuretic peptide; AVP, vasopressin. L-NMMA infusion was sustained for 60 minutes. P_within_ represents the probability of an effect of NOS inhibition after each treatment assessed by Friedman’s ANOVA. P_between_ represents the probability that paricalcitol alters the response to L-NMMA assessed by a general linear model for repeated measures with treatment as factor.

* = p < 0.05 vs. placebo at baseline.

## Discussion

The main objective of this randomized, placebo-controlled, crossover trial was to investigate the effects of paricalcitol on PRC with employment of diet standardization and a run-in phase where RAS inhibitors were discontinued. We found no significant effect of paricalcitol on PRC and 24-h AER, although AER during ^51^chrom-EDTA clearance was significantly lower in the paricalcitol group. Additionally, this study found that paricalcitol 1) blunted the albuminuric response to NOS inhibition, 2) increased serum FGF23 and 3) reduced GFR measured by ^51^Cr-EDTA clearance.

In the present trial, paricalcitol treatment did not affect plasma concentrations of renin and AngII significantly. Aldo tended to increase which may have been caused by slightly higher plasma potassium during paricalcitol treatment. Suppression of RAS is thought to be an important mediator of the reno-protective properties of VDRAs in CKD [17]. Most of our current knowledge regarding the relationship between vitamin D and RAS originates from animals studies, where it has been shown that calcitriol down-regulates renin expression independently from PTH and calcium levels [18]. Renin expression is highly elevated in VDR null mice, which leads to systemic hypertension, cardiac hypertrophy and heart failure [19]. Although an inverse relationship between 1,25(OH)_2_D and PRC has been documented in humans [20], prospective clinical trials have not confirmed this relationship satisfactorily. The results of a descriptive study of 17 children and young adults with hereditary vitamin D resistant rickets were remarkable, as none of the patients had hypertension, left ventricular hypertrophy or PRA elevation [21]. In the VITAL study paricalcitol did not suppress PRA in patients with diabetic nephropathy, although albuminuria was reduced [4]. Thus, the role of vitamin D in the complex regulation of renin expression remains to be established in humans, and ultimately raises the question whether a causal relationship between vitamin D and renin secretion exists. Eraranta et al. proposed that an answer may lie in the timing of vitamin D treatment in relation to renal injury [22]. In 5/6 nephrectomized rats, Freundlich et al. documented a significant suppression of renin-angiotensin genes when paricalcitol was initiated a few days after surgery [5]. On the contrary, instituting paricalcitol treatment 15 weeks after 5/6 nephrectomy did not cause any changes in RAS components [22,23]. The latter animal model of CKD may more accurately resemble the state of chronic renal insufficiency in man.

Twenty-four-hour urinary albumin excretion was not significantly reduced after paricalcitol, although the decrease in AER during the 90-min baseline period of the clearance experiment was more pronounced and statistically significant. Thus, our results do not unambiguously confirm the findings from precious studies regarding the effects of paricalcitol on albuminuria [2-4].

Glomerular permeability to albumin is highly dependent on NO [10], which led us to explore the effect of paricalcitol on renal NO dependency by acute infusion of L-NMMA, a non-selective NOS inhibitor. The L-NMMA dose chosen in our study was based on a preliminary dose–response trial, in which hemodynamic and renal effects of L-NMMA were characterized [24]. In the present study, L-NMMA caused an acute increase AER when patients had received placebo. Paricalcitol, on the other hand appeared to blunt this increase in albuminuria, which could be caused by either prevention of the NOS inhibitor mediated increase in glomerular permeability, or by increased albumin reuptake. Tubular reabsorption of albumin involves cubilin and megalin [25], but the role of vitamin D in regulation of these receptors has not yet been elucidated. In patient populations prone to endothelial dysfunction, NOS blockade provokes albuminuria [11]. Moreover, flow mediated vasodilation correlates to the degree of albuminuria [26]. Endothelial NOS (eNOS) is expressed in endothelial cells, including those of the kidney. This enzyme plays an important role in maintaining endothelial function [27], which is illustrated by the progressive renal failure observed in eNOS knock-out mice [28]. A decrease in endothelial and renal NO production in CKD has been suggested to play a primary role in progression of kidney disease [19]. Little is known about the direct effects of vitamin D on renal NO bioavailability, although a few studies have indicated that vitamin D may play a role in the regulation of renal NO generation. In uremic rats paricalcitol restored eNOS deficiency [12], and calcitriol increased NO production in cultured endothelial cells [13]. Thus, it seems plausible that selective VDRAs may reduce proteinuria through favourable modulation of the NO system. However, the results from the current trial do not have very strong statistical confidence, and should be interpreted with caution.

Our study showed a marked rise in FGF23 after paricalcitol treatment, which is consistent with previous studies of VDRA therapy in both predialysis [29] and dialysis patients [30]. In CKD, elevated FGF23 has been associated with increased cardiovascular mortality, and the rise in FGF23 in early stages of CKD is believed to be a main contributor to the decline in vitamin D levels through the induction of CYP24A1 mediated degradation [31,32]. Whether FGF23 is truly an independent risk factor or merely a marker of other abnormalities in CKD remains to be established.

Although this study was not primarily designed to investigate the effects of paricalcitol on GFR, we found a modest but statistically significant 3 ml/min/1.73 m^2^ decline in ^51^Cr-EDTA clearance after six weeks of paricalcitol treatment compared with placebo. Relatively, the decrease in creatinine based eGFR was larger than the decrease in clearance based GFR measurements. Activated vitamin D compounds have long been known to increase plasma creatinine [33-37]. Hitherto alterations in creatinine based estimations of GFR have been attributed to increased tubular secretion of creatinine [37] as well as musculoskeletal effects of vitamin D in patients with uremic myopathy [34]. Inulin clearance in eight [37] and ten patients [34] did not yield any significant changes in true GFR despite increases in creatinine. The effect of paricalcitol on GFR was specifically investigated in a study of 16 patients with CKD stage III-IV [33]. In this study, seven days of oral paricalcitol caused a reversible increase in plasma creatinine and 24-h urinary creatinine excretion without reducing iothalamate or creatinine clearance significantly. The authors documented an initial decrease in serum urea nitrogen and urea excretion rate, which may suggest an initial anabolic effect of paricalcitol. Preliminary results from a yet unpublished randomized, placebo controlled study in 45 patients with type 1 diabetes and nephropathy did not show any significant decrease in GFR measured by ^51^Cr-EDTA clearance, although creatinine based eGFR was significantly lower after 12 weeks of paricalcitol treatment (Joergensen C, Tarnow L, Goetze JP, Rossing P. Vitamin D analogue therapy, cardiovascular risk and kidney function in type 1 diabetic patients with diabetic nephropathy – a randomized trial. 2012. Personal Communication. Cited in agreement with the authors). These studies found small but non-significant reductions in clearance based GFR. In the Primo trial, both creatinine and cystatin C based estimations of GFR were significantly reduced after 48 weeks of paricalcitol^36^. If vitamin D reduces albuminuria through mechanisms similar to ACE inhibitors, the reduction in creatinine and cystatin C base GFR calculations may reflect a true decline in GFR, which will have important implications for the management of CKD.

During baseline of the clearance experiment where patients underwent water loading, urine output and free water clearance were lower after paricalcitol. This was not accompanied by any significant changes in FE_Na_, FE_K_, AVP, BNP or urinary excretion of ENaCγ and AQP2 channels, reflecting unaltered net tubular transport of sodium and water. The reduction in GFR may explain the decreased urine output and in turn be responsible for part of the reduction in AER.

To our knowledge this is the first study to document the renal and vascular response to NOS inhibition in vitamin D treated patients. Other strengths include the randomized, double blinded, placebo-controlled design, 24-h BP monitoring, and the employment of diet and fluid standardizations. Limitations include small sample size, short treatment duration, and the potential for pharmacodynamic carry-over effects of paricalcitol. For ethical reasons antihypertensive medication was maintained. In order to minimize the effects of agents directly influencing RAS, ACE inhibitors and AngII receptor blockers were discontinued and antihypertensive medication kept unchanged during the study. However, other medications such beta-blockers, potassium sparing diuretics and calcium receptor antagonists inevitably affect RAS activity as well. Furthermore, the discontinuation of RAS inhibitors and slightly uneven gender distribution may limit the generalizability of the results.

## Conclusion

In conclusion, this study did not provide evidence that paricalcitol suppresses circulating components of RAS in patients with albuminuric stage III-IV CKD. Paricalcitol reduced AER during the clearance experiment but not in 24-h urine, and appeared to abrogate the proteinuric response to NOS inhibition. Thus, it is possible that paricalcitol may decrease albuminuria through RAS independent mechanisms including reduction in GFR and favourable modulation of the NO system.

## Competing interests

Abbott Laboratories A/S provided study medication and partial funding for the project. The results presented in this paper have not been published previously in whole or part, except in abstract format.

## Authors’ contributions

All authors have made substantial contribution in designing the study and collection of data. They have all contributed in writing the manuscript and have read and approved the final version.

## Pre-publication history

The pre-publication history for this paper can be accessed here:

http://www.biomedcentral.com/1471-2369/14/163/prepub

## Supplementary Material

Additional file 1: Table S1 Experimental procedure. Click here for file
