# Supplementary material for: Effect of paricalcitol on renin and albuminuria in non-diabetic stage III-IV chronic kidney disease: a randomized placebo-controlled trial
Source: BMC Nephrol. 2013 Jul 26;14:163. doi: 10.1186/1471-2369-14-163 (PMC3729723; doi:10.1186/1471-2369-14-163)
Supplement: Additional file 1: Table S1 — Experimental procedure. [file 1471-2369-14-163-S1.doc]

| **Table 1 |** Experimental procedure | | | | | | | | | | | | | | | |
| --- | --- | --- | --- | --- | --- | --- | --- | --- | --- | --- | --- | --- | --- | --- | --- |
|  | Day -4 Day -3 Day -2 Day -1 | | | | Examination day | | | | | | | | | | |
| Time |  |  |  |  | 08:00-  08:30 | 08:30-  09:00 | 09:00-  09:30 | 09:30-  10:00 | 10:00-  10:30 | 10:30-  11:00 | 11:00-  11:30 | 11:30-  12:00 | 12:00-  12:30 | 12:30-  13:00 | |
|  |  |  |  |  |  |  |  | baseline (p1-p3) | | | p4 | p5 | p6 | p7 |  |
| Diet | Standardized diet | | | | Fasting | | | | | | | | | | |
| Water | 35 ml/kg/day | | | | 175 ml every 30 minutes | | | | | | | | | | |
| Urine samples |  |  |  | 24-h urine | ↑ ↑ ↑ ↑ ↑ ↑ ↑ ↑ | | | | | | | | | | |
| Blood  Samples |  |  |  |  | ↑ ↑ ↑ ↑ ↑ ↑ ↑ ↑ ↑ | | | | | | | | | | |
| Blood pressure | 24-h ABPM |  |  |  | ↑ ↑ ↑ ↑↑↑↑↑↑↑↑↑ ↑ ↑ ↑ | | | | | | | | | | |
| Applanation tonometry |  |  |  |  |  |  | ↑ |  |  | ↑ |  | ↑ |  |  | |
| ^51^Cr-EDTA |  |  |  |  | |--*--------------------------------- infusion* -------------------------------------------------------------- | | | | | | | | | | | |
| L-NMMA |  |  |  |  |  |  |  |  |  | | ----- *infusion* ----- | | | | |  | |
| Experimental procedure during each of the two examination sessions at the end of the six week treatment periods. ABPM, ambulatory blood pressure monitoring; L-NMMA, NG-monomethyl-L-arginine; p1-p7, clearance period 1-7. | | | | | | | | | | | | | | | |
